# The State-Led Large Scale Public Private Partnership ‘Chiranjeevi Program’ to Increase Access to Institutional Delivery among Poor Women in Gujarat, India: How Has It Done? What Can We Learn?

**DOI:** 10.1371/journal.pone.0095704

**Published:** 2014-05-01

**Authors:** Ayesha De Costa, Kranti S. Vora, Kayleigh Ryan, Parvathy Sankara Raman, Michele Santacatterina, Dileep Mavalankar

**Affiliations:** 1 Dept of Public Health Sciences, Karolinska Insitutet, Stockholm, Sweden; 2 Indian Institute of Public Health, Gandhinagar, Gujarat, India; University of Alabama at Birmingham, United States of America

## Abstract

**Background:**

Many low-middle income countries have focused on improving access to and quality of obstetric care, as part of promoting a facility based intra-partum care strategy to reduce maternal mortality. The state of Gujarat in India, implements a facility based intra-partum care program through its large for-profit private obstetric sector, under a state-led public-private-partnership, the Chiranjeevi Yojana (CY), under which the state pays accredited private obstetricians to perform deliveries for poor/tribal women. We examine CY performance, its contribution to overall trends in institutional deliveries in Gujarat over the last decade and its effect on private and public sector deliveries there.

**Methods:**

District level institutional delivery data (public, private, CY), national surveys, poverty estimates, census data were used. Institutional delivery trends in Gujarat 2000–2010 are presented; including contributions of different sectors and CY. Piece-wise regression was used to study the influence of the CY program on public and private sector institutional delivery.

**Results:**

Institutional delivery rose from 40.7% (2001) to 89.3% (2010), driven by sharp increases in private sector deliveries. Public sector and CY contributed 25–29% and 13–16% respectively of all deliveries each year. In 2007, 860 of 2000 private obstetricians participated in CY. Since 2007, >600,000 CY deliveries occurred i.e. one-third of births in the target population. Caesareans under CY were 6%, higher than the 2% reported among poor women by the DLHS survey just before CY. CY did not influence the already rising proportion of private sector deliveries in Gujarat.

**Conclusion:**

This paper reports a state-led, fully state-funded, large-scale public-private partnership to improve poor women’s access to institutional delivery - there have been >600,000 beneficiaries. While caesarean proportions are higher under CY than before, it is uncertain if all beneficiaries who require sections receive these. Other issues to explore include quality of care, provider attrition and the relatively low coverage.

## Introduction

The prioritisation of the intra- partum period is central to any strategy that aims to reduce maternal mortality. A health centre based intra-partum care strategy has been recommended as an effective means of reducing high maternal mortality, as most maternal deaths occur during labour, delivery, or the first 24 hours post-partum, from complications that cannot always be predicted or prevented [1].

India adopted an institutional delivery strategy for intra-partum care under its National Rural Health Mission in 2005. In much of the country, this strategy was implemented through the Janani Suraksha Yojana (JSY) which is a conditional cash transfer paid out to women who deliver in a facility. The program is run largely in public sector health facilities. Although a process for accreditation of private facilities is available by policy, accreditation of private sector facilities has been low. A very low proportion of JSY beneficiaries, between 0.5% and 2.4% were reported to have had deliveries in accredited private facilities in the large Indian states, [2]. This is a small proportion, given that India has 30,000 qualified obstetricians [3] mostly practicing in the private sector [4].

In addition to JSY, there have been other innovative methods to implement the institutional strategy for provision of intra-partum care. Much less is known about the performance of large-scale facility-based strategies when routed through the private sector, particularly in the face of weak provision by the public sector. The public health sector in Gujarat, a large western province (population 60 million), has suffered from shortages of specialist qualified staff (obstetricians) to provide appropriate emergency obstetric care services. In 2008, only 21 of 56 public First Referral Unit (FRUs) facilities, responsible for the administration of comprehensive emergency obstetric care, had an obstetrician. This shortfall was even greater in the community health care centres (second tier public health facilities with 20–30 beds); only 6 of which had an obstetrician in the province. In comparison, during the same period there were about 2000 obstetricians working in the private sector [5].

Recognising the inadequacies of the public health care system to provide emergency obstetric care to pregnant women, and that women from vulnerable groups were most likely to require such care, the government of Gujarat launched “Chiranjeevi Yojana” (CY) in all districts in Jan 2007. The scheme is a Public Private Partnership (PPP), which aims to improve access to institutional deliveries for women from those groups that bear the highest burden of maternal mortality. Under this ‘performance based financing’ program, the state pays accredited private obstetricians an agreed fee for the performance of deliveries of poor and/or tribal women. Since full implementation of the CY began in 2007, until 2010 more than 600,000 deliveries have been conducted under the scheme in Gujarat.

This program is a demand side financing program; it uses explicit performance-based subsidies to motivate private sector obstetricians to provide institutional delivery services of a defined level of quality and at an affordable cost to disadvantaged women. There have been some reports of other similar programs from Kenya [6], Cambodia [7] and Bangladesh [8], however none of these have been designed and led by the state nor have operated on the same scale as the CY program. The Kenya program, which was a smaller voucher based program, showed improved access to facility based delivery for poor women. It also appeared to have a persistent effect, as women who had one delivery under the program, were more likely to have subsequent births in a facility. The CY program is a much larger program, operating at scale, financed and led by the state government. It differs somewhat from the other programs in that eligible women are not required to purchase vouchers, instead the private obstetric facilities where these women choose to deliver are reimbursed directly by the state, on production of the requisite paper work.

Even though the initial design of the program has been described [4], [9], [10], little is known about the actual performance of this innovative, state run, large scale programme. There have also been concerns about whether such a program could negatively influence the proportion of public sector deliveries, and provide an impetus to private sector deliveries.

This paper aims to assess the contribution of the CY program to institutional delivery and its influence on facility delivery in the public and private sector; in the context of rising institutional delivery in Gujarat. The paper also studies the coverage provided by the CY to the eligible population, particularly their access to caesarean section under the program.

The study provides information on the functioning of a large-scale state led and state funded facility-based strategy for intra-partum care when routed through private sector facilities. The results from this study have implications for the program itself, as well as for policy makers and program managers in other low-middle income settings with large private sectors looking for innovative ways to reduce maternal mortality.

## Method

### Study Setting

Gujarat, a large province on India’s western flank, is home to 60.1 million inhabitants, over half of whom live in rural areas (57.4%) [11]. The state is socioeconomically relatively better off than other Indian provinces, it ranks as India’s third richest state based on GDP per capita, though recent estimates suggest a third of the population lives below the poverty line [12]. Scheduled tribes or ST (these are special groups listed under Article 366 (25) of the Indian constitution. They are the indigenous groups, often forest dwellers, and are recipients of positive affirmative action after India’s independence in 1947) constitute 14.8% of the population [11]. The state has a literacy rate of 79.3% and the latest estimates for the maternal mortality and infant mortality stand at 148/100 000 (2007–2009) [13] and 38/1000 live births (2012) respectively [14].

The province is sub-divided into 26 administrative districts (median district population = 2.1 million). The proportion of ST varies greatly by district, (0.2% in Amereli to 93.8% in The Dangs) as does the proportion below poverty line (BPL) (19.5–72.5%). Each district has its own health administration, which reports program performance within its boundaries though all health programs are guided and funded by the provincial government health department.

### The Chiranjeevi Yojana

In December 2005 the government of Gujarat launched Chiranjeevi Yojana (CY) as a pilot program initially in five districts and then scaled up in all districts of Gujarat in January 2007 [15]. The program was initiated against a backdrop of a severe shortage of qualified obstetricians in the state’s public sector hospitals (only 37% of the secondary level care facilities had obstetricians). Public health facilities, which are normally free of cost, are used mostly by poor and tribal women, thus shortage of obstetricians affected this group the most adversely. This group of poor and tribal women suffers the highest burdens of maternal mortality. On the other hand, the availability of more than 2000 obstetricians working in the fee-for-service private health sector, motivated the state to set up the private-public partnership (PPP) based CY program and harness the skills available in the private sector to deliver maternal health outcomes to women belonging to weaker socio-economic groups. Under the CY, the district health department enters in contracts with private obstetricians who own hospitals where deliveries (including cesareans) can be done. Enrolled obstetricians are paid by the provincial government to perform the deliveries of poor or tribal women (each identified by documentation provided to these groups). Thus women living BPL or belonging to ST can receive cash-free deliveries at private facilities. The obstetricians are paid a fixed sum per 100 deliveries of eligible women conducted. The sum is based on an average cost per delivery assuming 85 normal deliveries and 15 complicated deliveries, including 7 needing cesareans per 100 deliveries. The fixed reimbursement per 100 deliveries was based on cost calculations made by a leading non-governmental organisation in the province, but consultations were also held with various stakeholders including local arms of professional Obstetric and Gynaecological Societies in the province. The fixed reimbursement system has an embedded disincentive for the performance of caesarean sections in excess of 7 per cent. After recent revisions, the current fixed reimbursement stands at Rs.380, 000 per 100 deliveries ($6150 at current exchange rates).

A private obstetric care facility needs to meet certain predefined criteria to be accredited under the CY. The facility must be run by a specialist obstetric doctor, who owns the facility, it must have at least 15 beds plus labour and operating rooms and must be able to access blood for transfusions and be able to arrange anaesthesiologists and emergency surgery. [16]. An enrolled CY provider is paid a stipulated amount for each batch of 100 deliveries, regardless of the obstetrician’s level of experience or the proportion of caesareans performed, or the size of town or city in which the private clinic is located.

### Data Sources

All data used for the analyses were secondary sources. All data sets were complete.

Five sources of data were used in this study:

- The Department of Health record of delivery data by provider (public, private, CY) in each of the 26 districts for the years 2001–2010. For the CY program specifically, annual data on delivery numbers, caesarean section (CS) and provider numbers was recorded annually. This data was used to describe the trends in institutional deliveries over the last decade, as well as to study the influence of the CY on public and private sector deliveries. CS proportions under the CY were also calculated from this dataset.
- The Census of India was used to provide the ST proportion [17]; literacy rate and rural proportion in the districts [11]. This was used to study the district characteristics that might explain variations in the CY uptake.
- The dynamic BPL list as updated on June 26, 2012 by the government. of Gujarat, [12] provided the proportion of BPL in each district. The BPL census 2002 was used to estimate the proportion of BPL families who were also ST. These data sets were used to calculate the eligible population for the CY program in each district.
- The District Level Household Survey 3 (DLHS-3) [18] was used to establish the proportion of deliveries which required a caesarean section from 2006–08.

### Definition of Outcome Variables: Institutional Delivery

The outcome of interest was the proportion of institutional deliveries (i.e. deliveries within a health facility) annually (2001–2010) in each district; categorised by the sector of care into four categories; total deliveries, private sector deliveries (non CY), public sector deliveries and deliveries under the CY program (2006–2010). Proportions for each category for each district and year were calculated.

### Definitions of Predictor Variables for Each District

#### Literacy rate

The definition of literacy used was the ability to read and write in any language.

#### Rural population

The rural population for each district was defined as the proportion of district population living in rural areas as defined by the Census of India.

#### ST population

The proportion of the population from ST in each district as obtained from Census of India 2001.

#### BPL population

The proportion of the population living below the poverty line (BPL) in each district was the dynamic BPL list updated on June 26, 2012 obtained from the state government website.

#### Eligible population

The proportion of ST families also registered as BPL was calculated using data from the BPL Census 2002. The eligible population proportion was estimated as being the sum of the ST and BPL proportions minus the proportion of BPL that were also ST.

Proportion of eligible deliveries conducted under CY: The total number of eligible deliveries (denominator) was estimated from the proportion of eligible population in each district. The proportion of eligible deliveries conducted under CY was then obtained by dividing the actual reported number of CY deliveries in each district by total number of eligible deliveries.

CY enrolled obstetricians: The absolute number of obstetricians enrolled in the CY programme as reported by the Department of Health for each year 2006–2010.

Proportion caesarean sections (CS) under CY: The absolute number of CS conducted under CY was obtained from the delivery data supplied by the Department of Health; this was used to determine the proportion of all CY deliveries that require CS from 2006–2010.

Proportion of CS in the general population: Data from the DLHS-3 survey was used to estimate the proportion of women in Gujarat reporting a CS birth by year from 2006–08.

#### Ethical approval

All data included in the study was aggregated secondary data available in the public domain. Ethical approval was obtained from the Institutional Ethical review board of Indian Institute of Public Health, Gandhinagar.

### Analysis

We calculated the proportion of deliveries from 2001 to 2010 in each district, stratifying by sector (public, private non CY, CY). We plotted it over time to evaluate the trend.

To study the influence of CY on the proportion of private and public sector deliveries, slopes for deliveries in the respective sectors pre and post CY were calculated, using mean regression carried out using generalized estimating equations to take into account the hierarchical structure of the data. Partial correlation was used to analyse the association between CY delivery proportions with districts background characteristics including ST, rural, literacy and BPL proportions.

STATA version 12 and R 3.02 were used for all the analysis.

## Results

### I. Trend in Average Institutional Delivery Proportions in the 26 Districts of Gujarat from 2001 to 2010

**Figure 1:**
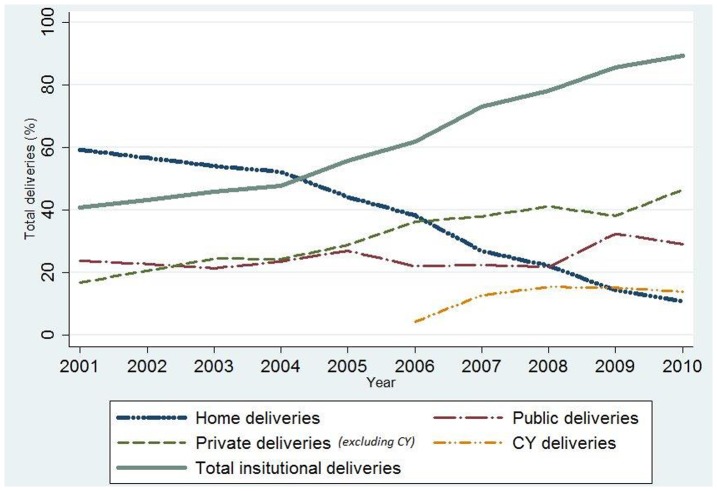
The trend in institutional deliveries by public sector, private sector and CY, Gujarat 2001–2010.

The total number of deliveries in Gujarat ranged from 1.0 to 1.2 million each year between 2001 and 2010. The average proportion of women giving birth in an institution more than doubled over that decade from 40.7% in 2001 to 89.3% in 2010 across the whole province (fig 1). The proportion of private sector delivery rose from 17% of all deliveries in 2001 to 60.3% in 2010– a very rapid and substantial increase. At the same time, public sector delivery proportions rose marginally from 24% in 2001 to 29% in 2010, indicating a large shift from home deliveries to private sector deliveries. The increase in proportion varied greatly by district (from 8% to 78%, data not shown). As seen in fig 1, the overall increase in total institutional delivery proportions was driven by a strong rise in private sector institutional delivery, by a moderate contribution from the CY program (after 2007) and a modest increase in public sector delivery proportion (24–29%).

### II. The Influence of CY Program on Delivery Proportions in the Public and the Private Sector

**Figure 2:**
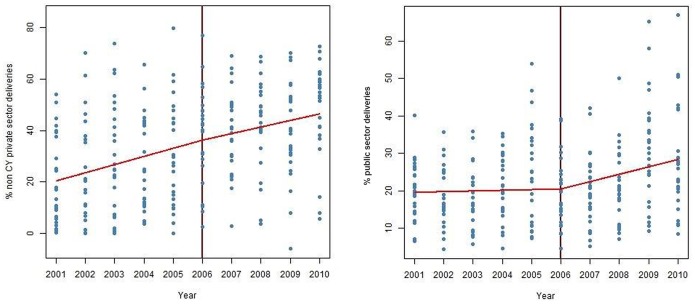
Mean regression (GEE) of proportion of (a) non-CY deliveries in private institutions (panel A) (b) public sector deliveries (panel B) over time in districts of Gujarat before and after CY.

Piecewise mean regressions of private (non CY) delivery proportions before and after CY are shown in figure 2a. While both the slopes (pre and post CY) are positive, indicating a rising private (non-CY) delivery proportion before and after the CY program, there was no significant difference between the two slopes pre and post CY (p = 0.32, coefficient = −0.62), indicating no statistically significant influence of the CY program on rate of increase of private institutional delivery proportions.

Piecewise mean regression for public sector delivery proportions before and after CY showed a rise in the slope of public sector deliveries after the CY (fig 2b). The slope for public sector deliveries post CY was more positive than pre CY, indicating some increase in public sector institutional delivery in the post CY period (p = 0.004, coefficient = 1.79).

### III. Coverage of Eligible Women in CY

**Figure 3:**
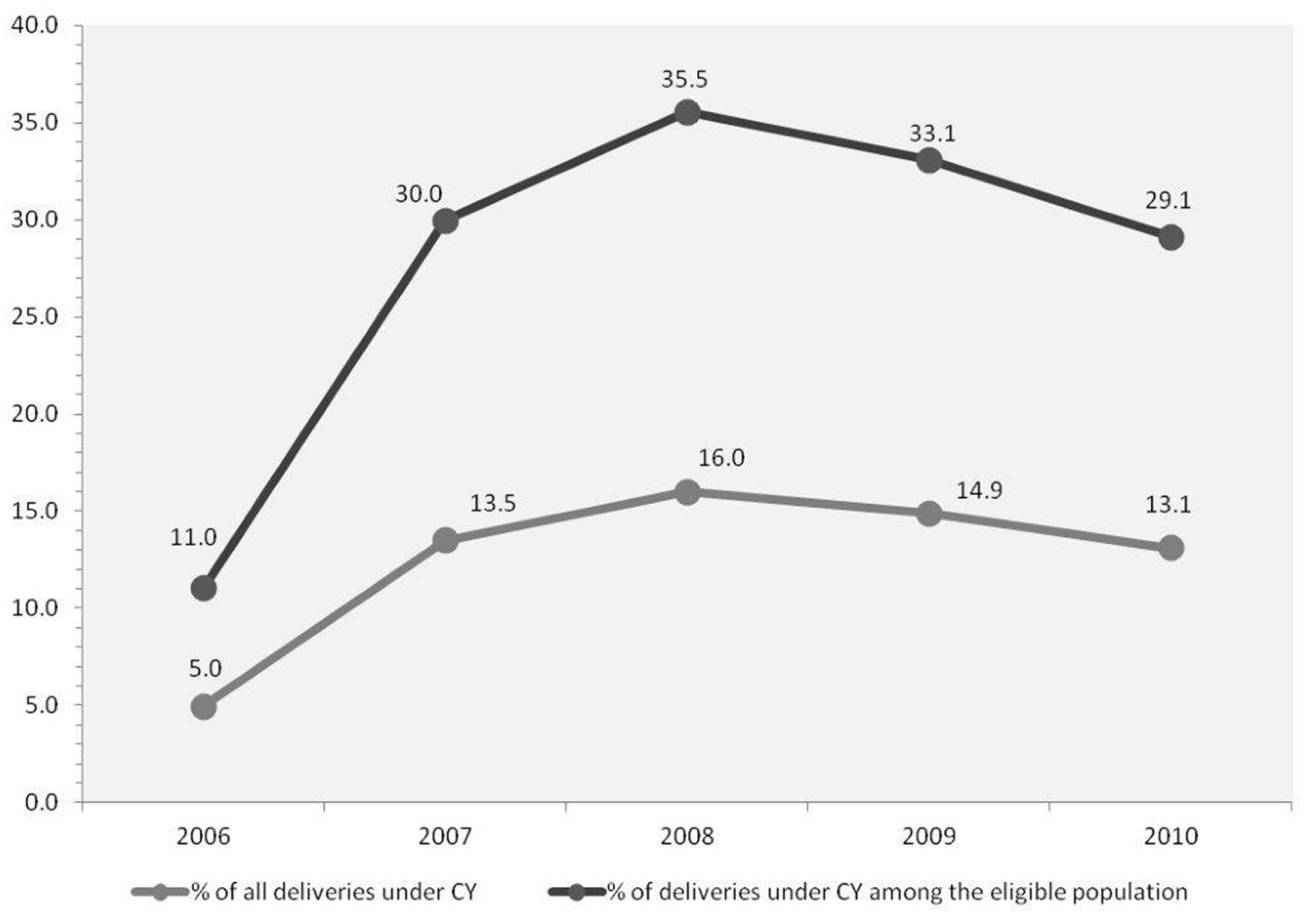
The mean percentage of deliveries conducted under the CY program, districts of Gujarat 2006–2010.

Between 2006 and 2010, a total of 624 612 deliveries had been conducted under the CY programme. Though the number of deliveries under the program is large, this has stayed steady between the years 2007–2010 (127,000–150,000 deliveries) as did the proportion of CY deliveries (13%–16%) as seen in fig 3. A small increase was seen in 2008. Among the eligible deliveries, i.e. BPL and/or ST women, the proportion of CY deliveries has also stayed steady at a third of all eligible deliveries, once again peaking at 35.5% in 2008 (fig 3).

Caesarean section proportions (CS) under the CY: Between 2006–10, 37,781 (6%) CY beneficiaries underwent CS delivery. This proportion of 6% is higher than the CS proportion in a comparable group of women (lowest two wealth quintiles from the District Level Household Survey (DLHS)-3 survey – (births between 2004–06) which reports only 2%. The DLHS-3 CS proportion could be reasonably considered indicative of a pre-CY estimate of CS proportion, therefore indicating an increase in CS proportions among the poor, under the CY.

### IV. District Level Variation in CY uptake, Provider Participation and Eligible Population

#### CY uptake

The proportion of CY deliveries between 2006 and 2010 varied widely between the districts ranging from 3.9% (n = 1372) of all deliveries in The Dangs to 32.8% (n = 81017) in Panchmahals (both poor tribal districts).

Private obstetricians participating and eligible population; The total number of obstetricians joining in the CY programme increased from a total 742 in 2006 to 867 in 2008, falling to 662 in 2010 (much of the provider attrition between 2008 and 2010 was from highly urbanized districts). The proportion of CY deliveries among the eligible population in the districts was not related to the density of CY providers in the district (per 100 000 eligible population), nor to the proportion eligible population in a district. In line with the increase in CY deliveries over time, the mean number of CY deliveries per CY obstetrician increased from 75 in 2006 to 259 in 2010; with wide variations between the districts. However CY uptake in 2010 was significantly associated with proportion rural population in the districts (partial correlation coefficient r = 0.42).

## Discussion

Gujarat like the rest of India has seen sharp increases in institutional delivery over the last decade. In the early part of the decade, the District level health survey reported in rise in national institutional delivery proportions from 34% in 1998 [19] to 41% in 2002 [20] to 47% in 2007 [21]. The initiation of the JSY program pushed institutional deliveries even higher, the Coverage evaluation survey (CES) by UNICEF in 2009 [22] reported 72% institutional delivery across the country, of which a third (26%) occurred in the private sector. Similarly, in Gujarat, DLHS-3 reported an institutional delivery proportion of 56.5% for Gujarat in 2007 and CES 2009 reported 78% institutional delivery. Importantly, more than half (44%) of these occurred in the private sector [22]. Gujarat has a strong network of private sector providers of institutional delivery services, mostly working on the basis of out-of-pocket payments from patients. The state ranks third among the large Indian provinces for proportion of private sector deliveries at 44%, behind the southern states of Andhra Pradesh and Kerala, each of which have 50% of institutional deliveries in the private sector. Our results indicate that private sector provision of institutional delivery services was much lower at the beginning of the decade (17% in 2001); however it has more than tripled in the province over the decade. At the end of the decade, most districts converged to have a high institutional delivery proportion with two thirds of all births occurring in the private sector.

The increase in the utilization of delivery care at private health centres may be attributed to many factors, including: a low level of trust in the care provided by public facilities, the availability of specialist physicians combined with perceived better quality of care and accessibility of private health centres, the non-availability of health services and drugs at public centres, the improvement in the standard of living of the overall population [23], [24]. In Gujarat, increased utilization may be due to a combination of the above factors, but it may also be in large part because the capacity in the public health sector to provide emergency obstetric care (including caesarean section) has been low. Previous reports have indicated a severe disparity in the number of qualified obstetrician/gynaecologists working in the public and private sectors [25].

### CY Coverage

There have been over half a million beneficiaries in first four years of the program; the proportion of births in the target population under the program has been steady at a third of all eligible deliveries since 2007 (first year of full implementation). While this is an encouraging result, it must be noted that the DLHS 3 data (data on births between 2004–2006), which can be considered a ‘baseline’ prior to the inception of the program reports the proportion of institutional delivery in the specific target group (BPL and ST) as being low (at 37.8% and 24.9% respectively). Coverage of this target group under CY has been similar, i.e. a third of all eligible beneficiaries. It is uncertain if the CY has provided additional coverage to previously uncovered women (i.e. women who might have otherwise delivered at home) or if it has shifted beneficiaries from services they were previously using (i.e. women who would otherwise have delivered in public institutions or in private institutions by paying fees) into the program. It is unlikely that a large fraction of women delivering in government facilities have moved to CY as proportion of deliveries in the public sector has not declined, on the contrary it has increased. However, the other two alternatives i.e. home deliveries and possibly private sector fee paying poor women, shifted into the CY – in both cases it is a positive outcome as access to institutional delivery has increased with low costs. The total number of CY deliveries has varied little and its contribution to overall institutional delivery is starting to dip. This needs to be further explored. It is possible that the level of political commitment to the program has waned, or that the program has seen attrition of private obstetricians, or both. It is not clear why CY deliveries did not increase beyond 35%, even when there were so many providers and deliveries were free to the beneficiaries. Though the mean uptake of CY among the eligible population is 33%, it varies widely between different districts. A reason for low uptake could be lack of awareness of the program among eligible women, partly because of deficient information dissemination strategies. This lack of awareness further extends to lack of knowledge on how to assemble the requisite paperwork to be presented in the facility [26]. The geographic distribution of CY providers also possibly plays a role in influencing uptake. The majority of CY providers are in bigger towns (district and block headquarters) and not in the hinterland, rural areas do not have a strong private obstetric care sector. Yet, CY uptake is significantly related to the proportion of rural population in a district, indicating that rural mothers belonging to these vulnerable groups are willing to travel to receive emergency obstetric care, even if the facility is some distance away. This travel was facilitated by the initiation of an emergency transport service provided free of cost to the user under the another public private partnership, [27] which started during at the same period as CY.

The recent attrition of private providers participating in the program could also be contributing to lower uptake. Most of the attrition has been from the large urban districts, where there are many other well-functioning alternatives (public and private) available for women to choose a place of delivery. It is possible that in these large cities or towns, CY providers were unable to attract enough women to deliver under the program for their participation to be viable. However the lack of relationship between CY uptake and density of CY providers or proportion eligible population in a district suggests that other factors such as location and availability of alternative emergency obstetric services may play a role in determining uptake. Monitoring issues, deficient planning and lack of buy in from all stakeholders have been quoted as some of the reasons which could result in low coverage of the CY, and limit its success [25].

### Caesarean Sections Under the CY

The program had a relatively higher caesarean section proportion (6–8%) among beneficiaries in comparison to the low rates (2%) reported by the DLHS-3 [18] among a comparable group of vulnerable women (the lowest two wealth quintiles) prior to the inception of CY. Increase in the caesarean section proportion among beneficiaries reflects the influence of the program in increasing access to comprehensive emergency obstetric care, which was one of the objectives of CY. Caesarean section proportion has increased from 2% in DLHS-3 to 6% in our data, but the rate among beneficiaries is still lower than the overall Caesarean -section rate in the private sector (18%) [28]. The financial reimbursement package for private obstetricians under CY was designed to limit unnecessary caesareans (embedded dis incentive for unnecessary caesarean) thus providers were paid a fixed amount per 100 deliveries, assuming a 7% caesarean proportion per 100 deliveries. This embedded disincentive had probably contributed to keeping the caesarean rate low, though it is not possible to conclude from the data whether only unnecessary caesareans were avoided. It may be possible that some of the providers are avoiding high risk women or refer complicated cases because of the fixed payment system.

### The Influence of the CY on Public and Private Sector Institutional Delivery

The 5^th^ Common Review Mission Report for National Rural Health Mission, published by the Government of India suggests that the CY could have negatively influenced the proportion of public sector deliveries in Gujarat [29] **.** Our study found that despite being implemented through the private sector (in the absence of a strong obstetric care public sector), CY has not reduced public sector institutional delivery proportions; on the contrary an increase in public sector deliveries has been noted towards the end of the decade – after initiation of the CY program. This is likely to be because of the JSY program which began at the same time, rather than any influence from the CY itself. While it is important to strengthen the public sector to deliver obstetric care of adequate quality, and efforts towards this must continue in the future, it is clear from the data that the CY has not contributed to a decline in public sector institutional delivery. Whether public sector delivery would have risen further, and by how much, in the absence of CY, is uncertain. However for most poor women, the CY program offers the possibility of accessing better levels of emergency obstetric care than they would receive in the public sector. Similarly CY did not contribute to a spurt in private sector deliveries. Private sector deliveries were rising sharply even before CY began; the program did not change the rate of increase. Reasons for the modest increase in public sector deliveries could include the fact that state and national policies promote institutional deliveries in general; especially through a new cadre of health volunteers called ASHAs (female community based Accredited Social Health Activists). The success of some other interventions by the Gujarat government, such as training general doctors for emergency obstetric care including CS and anaesthesia [26] could also have contributed. The increasing popularity of the 108 emergency transport (ambulance) service to transport mothers to hospital for delivery has also possibly been responsible [30], [31]. The lack of private providers in some rural areas could mean that for many mothers, the public sector is still the most feasible/accessible site for delivery. The incentives provided under the JSY, in the state though relatively small, (Rs 500–700, 8–12 weeks before delivery) [32] could also promote the on-going use of public sector facilities.

### Implications Beyond the Setting

There have been a number of demand side financing programs for maternal health that have recently been implemented in the global south, particularly in South Asia. The large national government funded cash transfer programs to promote institutional delivery in India [33] and Nepal [34] have been implemented largely through the public sector. There have been some demand side programs with similar objectives that have been implemented through the private sector as well, these have largely been voucher based programs in Cambodia, Bangladesh, Pakistan and Kenya [35]. These have been limited to smaller subnational areas and have often been funded by development partners interested in demand side financing initiatives. While there has been increased uptake of services reported under these initiatives, the CY is unique in that it is the first demand side financing program that is state driven, fully state funded (no external aid) and operating at scale, across a province of 60 million people. To our knowledge, this is the first paper reporting the performance of the program and the impact it has had on the public and private sector institutional delivery and CS rate. The CY, though not strictly a voucher based program, operates in the context of a strong private for-profit obstetric sector, a scenario seen in many low-middle settings. The private obstetric sector though strongly present, tends to be concentrated in cities and small towns, and does not extend into the rural hinterlands (very remote and poor areas). Though access is still difficult for some rural women, the program has brought functional emergency obstetric care closer to the beneficiaries. The program has succeeded in providing a degree of access to institutional delivery to its target group of vulnerable women, though steps to improve coverage and to manage private provider attrition need to be looked into. The proportion of caesareans among this group rose to 6% under the program, in comparison to earlier lower levels of 2%. The embedded disincentive for caesarean section has possibly prevented caesarean proportions from rising particularly sharply, which has been known to happen when CS are differentially reimbursed, however we do not know whether all vulnerable women who require caesarean receive this. This will require a more in depth investigation.

### Methodological Considerations & Limitations

Data for institutional delivery and CY program statistics were available at the district level; hence the district was used as the unit of analysis. Sub district level statistics are collated at district level and then reported at state level from where this data was obtained. Though the data is complete, a limitation of this study is the fact that data used in this study was collected for health information and monitoring purposes. There are, therefore unanswered questions that could have been answered if data specific to the CY program had been collected at the appropriate time and in the appropriate population strata. However the analysis of this dataset itself yields valid conclusions.

The proportions of eligible population, i.e. ST and BPL were obtained from the Census India 2001 and BPL Census 2002 respectively. More recent ST estimates from the 2011 census are not yet available in the public domain. While BPL proportions are available, the measurement criteria have changed, rendering a comparison with previous estimates difficult. This analysis therefore assumes that the proportions of the eligible population in the district are constant over the last decade.

### CY Providers

Only annual total numbers of CY providers were available. As individual providers cannot be identified, we are unable to establish the details of how many new providers joined and how many left the programme each year. Also as there exists no reliable number of the potential providers who could have joined the program each year, there is no denominator for the participation of CY providers, and the proportion of eligible providers that joined the programme cannot be ascertained.

## Conclusions

The results reported in this paper provide the first review of the performance of a large scale program to increase access to intra-partum care that has been routed through the private sector under state stewardship and state funding. The CY program accounted for 15% of all deliveries in the state and provided access to emergency obstetric services for a third of all eligible mothers though uptakes, which varied across districts. Caesarean proportions were higher under the program than the pre-program estimates. The program has not influenced already rising private sector facility delivery proportions in the province. These findings are of relevance to the program as well as to policy makers/program managers in low middle income settings with large private sectors, looking to promote a facility based intra partum strategy. The results from our study also indicate directions for further research in the CY program and other such large public private initiatives. These include differences in outcomes between program users and non-users, quality of care issues (particularly important in maternal health), and further exploration of reasons behind differences in district level uptake of the CY, including studies of the location of facilities using a geographic information system. Different strategies to ensure private provider retention when such partnerships operate at scale need to be tested. The paper also indicates the importance of the collection of routine (and program) data on institutional delivery disaggregated by population sub-groups.
